# The Geriatric Emergency Department Intervention model of care: a pragmatic trial

**DOI:** 10.1186/s12877-018-0992-z

**Published:** 2018-12-03

**Authors:** Marianne Wallis, Elizabeth Marsden, Andrea Taylor, Alison Craswell, Marc Broadbent, Adrian Barnett, Kim-Huong Nguyen, Colleen Johnston, Amanda Glenwright, Julia Crilly

**Affiliations:** 1 School of Nursing, Midwifery and Paramedicine, University of Sunshine Coast, 90 Sippy Downs Drive, Sippy Downs, QLD 4556 Australia; 2 Sunshine Coast and Hospital Health Service, Emergency Services, Birtinya, QLD Australia; 3 Institute of Health and Biomedical Innovation & School of Public Health and Social Work, Queensland University of Technology, Kelvin Grove, QLD 4059 Australia; 4 Center for Health Service Research, Faculty of Medicine, University of Queensland, Herston, QLD 4006 Australia; 5 Program Management Office, Central Queensland, Wide Bay, Sunshine Coast PHN, Ground Floor, Mayfield House, 29 The Esplanade, Maroochydore, QLD 4558 Australia; 6 Menzies Health Institute Queensland, Griffith University, Gold Coast Campus, Griffith, QLD 4222 Australia; 7 Department of Emergency Medicine, Gold Coast Health, Southport, QLD Australia

**Keywords:** Geriatric, Emergency medical services, Nurses’ practice patterns, Hospital, Homes for the aged, Delivery of health care, Protocol, Outcomes, Evaluation, Pragmatic paradigm

## Abstract

**Background:**

To evaluate a Geriatric Emergency Department Intervention (GEDI) model of service delivery for adults aged 70 years and older.

**Methods:**

A pragmatic trial of the GEDI model using a pre-post design. GEDI is a nurse-led, physician-championed, Emergency Department (ED) intervention; developed to improve the care of frail older adults in the ED. The nurses had gerontology experience and education and provided targeted geriatric assessment and streamlining of care. The final format included 2.4 full time equivalent nurses working 7 days from 0700 h to 1730 h (1530 h at weekends). There were three implementations periods: pre-implementation (2012); a developmental phase from January 2013 to August 2015; and full implementation from September 2015 to August 2016. The outcomes measured were disposition (discharged home, admitted or died); ED length of stay; hospital length of stay; all cause in-hospital mortality within 28 days; time to ED re-presentation up to 28 days post-discharge; in-hospital costs.

The setting was a tertiary hospital ED, with 385 beds, in Queensland, Australia. Approximately 53,000 patients presented to the ED annually with 20% aged 70 years and older. All patients over the age 70 who presented to the ED between January 2012 and August 2016 (*n* = 44,983) were included in the trial.

**Results:**

Older persons who presented to the ED when the GEDI team were working had increased likelihoods of discharge (Hazard ratio (HR) = 1.19; 95% CI: 1.13–1.24) and reduced ED length of stay (HR = 1.42; 95% CI: 1.33–1.52) compared with those who presented when GEDI were not working. There was no increase in the risk of mortality (HR = 1.01; 95% CI = 0.23–4.43) or risk of same cause re-presentation to 28 days (HR = 1.21; 95% CI: 0.99–1.49). The GEDI service resulted in average cost savings per ED presentation of $35 [95% CI, $21, $49] and savings of $1469 [95% CI, $1105, $1834] per hospital admission.

**Conclusions:**

Implementation of a nurse-led physician-championed model of ED care, focused on frail older adults, reduced ED length of stay, hospital admission and if admitted, hospital length of stay and cost, without increasing mortality or same cause re-presentation. These increases were sustained over time and after the initial implementation team had changed roles.

**Trial registration:**

Australian Clinical Trials Registration Number ACTRN12615001157561 - retrospectively registered on 29/10/2015. Data were retrieved via retrospective access to clinical information systems. First data access was on 1/7/2015.

## Background

As a consequence of the ageing of the population, older people comprise an increasing proportion of emergency department (ED) presentations [1]. Many frail older persons live in residential aged care facilities (RACFs) but 30 to 50% live in the community [2, 3]. When presenting to the ED, frail older adults receive a greater number of tests, spend longer in ED awaiting disposition planning and are at greater risk for medical complications, functional decline and poorer health following discharge [4, 5]. If RACF residents are hospitalised they suffer higher rates of adverse events and are susceptible to de-conditioning and worsening cognitive state [6–8]. Hospital admission for frail older adults is associated with increased morbidity and mortality [9, 10].

Previous studies have indicated ways in which the care of frail older adults experiencing acute illness can be improved [11, 12]. There are numerous suggestions for improvements in RACFs focusing on enhancing primary care [1, 13–15]. In the ED there are several studies looking at older persons over the age of 65, however, these actively exclude RACF residents [16, 17]. The limited previous research on this topic indicates that to improve the care of frail older adults in the ED there should be a single point of contact and structured communication tools [12, 17]; advanced aged care assessment at point of ED entry [11, 12, 17–19]; expert gerontological care by both medical and nursing staff [11, 20, 21]; and streamlining of patient management in ED [17, 22, 23]. The increased costs of these additional services means that research in this area must also collect data on costs as well as effectiveness.

Clinicians at a regional hospital in Queensland, Australia in collaboration with staff from a local RACF identified that both RACF residents and frail older adults living in the community were experiencing high rates of ED presentation and having worse outcomes than other cohorts. In consultation with university colleagues and the local primary health network (PHN) they designed the Care coordination through Emergency Department, Residential Aged Care and Primary Health Collaboration (CEDRiC) project. This was a two-pronged model of service delivery with interventions in both the RACF and ED. In the ED the Geriatric Emergency Department Intervention (GEDI) was implemented while a Nurse Practitioner Candidate was introduced to the aged care facility.

## Methods

The aim of this study was to evaluate the effectiveness and cost of the GEDI model of service delivery for adults aged 70 years and over, presenting to an ED in regional Queensland, Australia. The protocol for the structure, process and outcome evaluation [24] of the GEDI model has been published [25].

A randomised trial of individual patients was not feasible when considering a change to the model of care for a whole ED, and a cluster-randomised trial would be costly. Hence we undertook a pragmatic trial using a pre-post design that tracked eligible patients before and after the intervention was implemented [26].

### Participants and data collection

De-identified data for all patients aged 70 years and older, who presented to the study ED from January 2012 through to 31st August 2016, were retrieved from Emergency Department Information Service (EDIS; Healthcare Group, CSC)® and Hospital Based Corporate Information System (HBCIS; iSoft) databases. Data extraction was undertaken by health service data managers with deterministic linking of the EDIS, HBCIS and financial data. There were three time periods:

1. 1st January to 31st December 2012: prior to development of any aspect of GEDI (hereon referred to as – “pre-intervention or pre-GEDI”);
2. 1st January 2013 to 31st August 2015 – the development phase of the intervention during which funding and staffing models changed (hereon referred to as – “interim intervention or interim GEDI”); and
3. 1st September 2015 to 31st August 2016 – (hereon referred to as “full intervention”).

To overcome the ‘improvement-evaporation effect’, whereby the benefits reaped from new practices diminish over time [27] we used data from the immediate period post intervention (interim intervention), when the model was being developed and staffed by the most experienced and dedicated clinicians, but also included the full implementation period which represents a more standard implementation environment.

Independent variables that were used to describe the sample and to build multivariable models to compare outcomes were:

- Demographics - Age, Sex
- Date and time of presentation
- Clinical diagnosis – reason for presentation as ICD-10 code – this variable was then mapped to 25 major diagnostic categories
- Arrival by ambulance
- Presented from RACF
- Australasian Triage Score (ATS) [28, 29] – three groups 1&2, 3, 4&5
- Adult Deterioration Detection System (ADDS) Score [28]
- Time (per 100 days increase across the project)
- Presented to ED during GEDI working hours - Yes/No
- Intervention group (explained below)

### Intervention

The intervention has been described previously [25, 30] and is detailed within an Implementation Toolkit [31]. The aims of the model of care are to avoid hospital admission, if appropriate, and where this is not possible, to fast-track admission and medical management. Briefly, the GEDI intervention is delivered in the ED by a multidisciplinary team consisting of an ED physician champion and advanced practice ED nurses who have at least 5 years of experience working with older adults and preferably post-graduate qualifications in gerontology. The GEDI nurses operate as a supplementary sub-speciality team assisting the primary ED nurses and physicians. They target all patients over the age of 70 years especially those transferred from RACFs. They will either receive referrals from the primary care team or will identify patients via the electronic medical records system or via routine rounding. The use of frailty screening was trialled but was not found to be of use for these experienced clinicians.

In consultation, with the ED physician champion they will undertake targeted geriatric assessment (using the aspects of a comprehensive geriatric assessment that are appropriate for the individual patient) and problem formulation. They will then work with the primary ED team to fast-track diagnostic processes and engage the multi-disciplinary team and, where possible, the family in client-centred decision-making. Where necessary GEDI will undertake early referral to specialist care and/or activate standardised fast track pathways e.g. for orthopaedic surgery or stroke management.

When necessary, GEDI will coordinate admission to a specialty in-patient ward avoiding, where possible, a stay on a medical assessment unit. When admission is not required, the GEDI team may assist the primary care team with hands on care (e.g. wound care, catheter change etc.) and/or liaise with appropriate community or RACF services, to mobilise resources within the patient’s home to ensure safe discharge. Finally, the team communicates all ED care and future requirements of care to either the ward or the community care team, including the GP.

The GEDI team also provides an ongoing staff development program for other ED staff. Through a program of in-service education sessions new and junior staff are provided with information about the model of care and are also educated about geriatric syndromes, cognitive assessment and the care pathways used in the department.

### Objectives

The study objectives were to test differences in disposition, ED and hospital length of stay, time to ED re-presentation, all-cause mortality and costs between the groups of patients who presented, to the study ED, in the three time periods.

### Outcomes

#### Primary

- Disposition - discharged home, admitted, died

#### Secondary

- ED length of stay– in minutes
- Hospital length of stay – in days
- All cause in-hospital mortality within 30 days of ED presentation
- Time to ED re-presentations up to 28 days
- Cost of hospital admission

### Patient and public involvement

This project and the development of the intervention were a co-design activity that included two members of the public who were over 70 years of age. They are volunteers who spent a lot of their time visiting older adults in aged care facilities and had both personal and proxy experience of the health service and the ED. They not only joined the External Advisory Group set up to guide the development of the intervention and the conduct of the study but also visited the ED and a local aged care facility with members of the research team to discuss issues specific to older adults.

### Statistical methods

An independent statistician, not otherwise involved in the study, provided statistical summaries and analysis. Descriptive statistics were used to describe the participants in the three time periods including frequencies, percentages, appropriate measures of central tendency and distribution.

For the primary outcome, survival analysis was used to jointly model length of stay and disposition, with the three destinations as competing risks [32]. We used survival analysis for ED re-presentations with out-of-hospital mortality as a competing risk. All models adjusted for the patient level factors of gender, age, ATS, season, day of the week and time of presentation. Survival analysis is ideal here because our primary outcomes are times that are subject to censoring from competing risks. Previous analysis have simply categorised time and used logistic regression, for example discharge within 24 h. However, this wastes valuable information and reduces statistical power. We used cumulative incidence curves to look for changes due to the intervention over all times.

Pre-post designs are vulnerable to confounding by other changes over time that may be attributed to the intervention [33]. To control for this, we included a linear trend (based on date) in all models to account for gradual changes that are not captured in the individual variables, e.g., experience of healthcare workforce. We also adjusted for season using a sinusoid with an annual cycle to control for the winter peak in morbidity [34]. The survival analyses used Cox proportional hazard survival models. The models’ residuals were checked for outliers and correlation over time. We calculated Cook’s influential statistic and examined relatively large outliers. We calculated the variance inflation factor and removed variables with a score above five on the basis that they were co-linear. The key outcome was the mean effect of the intervention together with 95% confidence intervals.

We compared patients who may have received the GEDI intervention to patients for whom it was not available. This “usual care” group could be those who were admitted before the intervention was introduced, or those who were admitted after the intervention but outside the GEDI team’s hours. We also expected the intervention to change during the interim and full period, hence we created five categories:

- Pre intervention (control)
- Interim intervention during GEDI working hours (intervention)
- Interim intervention outside GEDI working hours (control)
- Full intervention during GEDI working hours (intervention)
- Full intervention outside GEDI working hours (control)

Hence we had three control groups and two intervention groups. We used the additional control groups because we suspected that there would be carry-over effects from the intervention that may create some additional benefit compared with the pre-intervention controls.

### Economic analysis

We compared the hospital lengths of stay and costs for the three time periods. We specified a two-stage recursive model which exploits the unidirectional causal pathway among the endogenous outcome variables (i.e. length of stay and cost) such that, for a given set of exogenous variables, the endogenous variables can be identified sequentially [35]. The exogenous variables that potentially influenced the length of stay and cost include: patient individual characteristics (age, gender and place of residence), the properties of the presentation (mode of arrival, major diagnostic category and mode of discharge) and whether or not the patient arrived during working hours. Since the recursive model does not allow variables that are linked in a causal chain to have correlated with the error terms, it can control for unobserved heterogeneity and endogeneity within the data [35, 36].

## Results

A total of 44,983 records were retrieved (pre-GEDI *n* = 9066; interim GEDI *n* = 25,675; full-GEDI *n* = 10,242). The patients’ mean age was 81 years (sd = 7) and 51% were female. Most presentations were on weekdays (with peaks on Mondays and Fridays) and between 0800 and 1900 h. There were no important differences between the three time periods for these variables (See Table 1).

**Table 1:** Demographic and clinical characteristics of the sample

Characteristic	Pre-implementation Group n = 9066	Interim-implementation Group *n* = 25,675	Full-implementation Group n = 10,242
Age - mean (sd)	81 (7)	81 (7)	81 (7)
Male gender – n (%)	4349 (48)	12,543 (49)	5073 (50)
Presenting conditions – n (%)			
Cardiac	2441 (27)	6586 (26)	2439 (24)
Trauma	1557 (17)	4674 (18)	1860 (18)
Gastrointestinal	828 (9)	2259 (9)	914 (9)
Respiratory	805 (9)	2102 (8)	883 (9)
Neurological	735 (8)	2221 (9)	865 (8)
Other (20 codes)	2696 (30)	7830 (30)	3273 (32)
Missing	4 (< 0.1)	3 (< 0.1)	8 (< 0.1)
Arrived by ambulance- n (%)	7196 (79)	19,830 (77)	7746 (76)
Australian Triage Score Category – n (%)			
Resuscitation	139 (2)	406 (2)	146 (1)
Emergent	2521 (28)	6968 (27)	2554 (25)
Urgent	4219 (47)	12,040 (47)	4754 (46)
Less urgent	2043 (23)	5776 (23)	2581 (25)
Non-urgent	144 (2)	485 (2)	207 (2)

N.B*.* Some column percentages do not add to 100% because of rounding

There was a small difference in the major diagnostic categories attributed to the three groups with the full intervention sample having a smaller proportion of cardiac presentations (*p* < 0.001), the Interim group having fewer presentations for respiratory conditions (p < 0.001) and the pre-intervention group having fewer presentations for trauma (p < 0.001). More patients arrived by ambulance in the pre-intervention and interim periods than during the full intervention (p < 0.001). Also there were between group differences in ATS category with the full intervention group having a larger proportion in ATS category 2 (urgent, see within 10 min) compared to the other time periods (p < 0.001). The differences between the three groups noted above were relatively small, and we attempted to minimise the impact of these differences by adjusting for the above variables in our survival models.

### Outcomes

#### Primary outcome

**Fig. 1:**
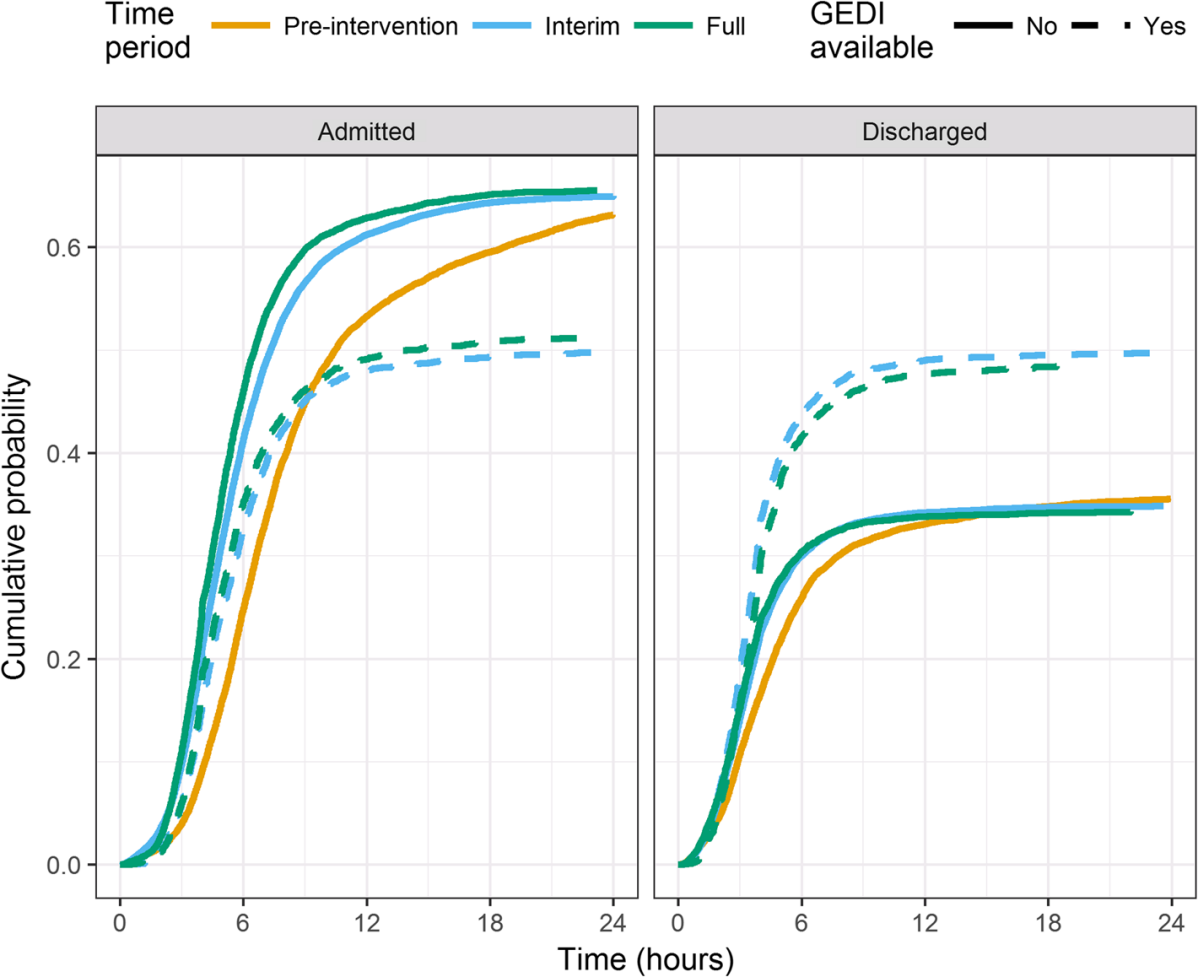
Cumulative incidence of admission or discharge in the first 24 h after presentation to ED for the five intervention groups. Results are not adjusted for potential confounders

The total follow-up time was over 246,000 days. Patients who presented during the GEDI intervention periods, during the GEDI working hours, were more likely to be discharged (See Fig. 1). After 24 h, the discharge probability for the two intervention groups was close to 0.50, whereas the three control groups were closer to 0.35 (See Fig. 1). The adjusted hazard ratios, for discharge of patients presenting in the GEDI intervention periods, ranged from 1.19 (full intervention during GEDI working hours, 95% CI 1.13, 1.24) to 1.31 (interim intervention outside GEDI working hours, 95% CI 1.23, 1.39). As expected, many of the patient characteristics influenced discharge, with older patients and those arriving by ambulance less likely to be discharged. There was also a slight trend over time for increased discharge (See Table 2).

**Table 2:** Hazard ratios and 95% confidence intervals for discharge using a Cox survival model with 5 GEDI groups

Predictor	Hazard Ratio	95% CI
Time (per 100 days increase)	1.02	1.02, 1.03
Age (per 10 year increase)	0.95	0.94, 0.97
Male sex	1.01	0.99, 1.04
Arrival by ambulance	0.72	0.70, 0.75
Australian Triage Score 1 and 2	0.94	0.90, 0.97
Australian Triage Score 3	0.82	0.80, 0.85
Presented from RACF	0.92	0.89, 0.95
MDC Diagnosis = Cardiac	0.95	0.92, 0.97
MDC Diagnosis = Trauma	1.22	1.18, 1.25
GEDI group (primary outcome):		
Interim outside GEDI hours	1.31	1.23, 1.39
Interim during GEDI hours	1.20	1.11, 1.29
Full outside GEDI hours	1.47	1.41, 1.53
Full during GEDI hours	1.19	1.13, 1.24

*NB* Reference for GEDI is pre-intervention. Reference for ATS is 4&5. Reference for diagnosis is all other matched diagnostic codes

Legend: *MDC* Major diagnostic category

There was a reduction in length of hospital stay, for admitted patients, of approximately 1 day between the pre-GEDI time period and the two GEDI intervention periods (Table 3).

**Table 3:** Comparison of Hospital length of stay (days) by GEDI intervention period using mean differences, 95% confidence interval for the mean difference and *p*-value from an unpaired t-test

Comparison	Mean difference	95% CIs	P-value
Pre vs Interim	−0.96	−1.02, − 0.90	< 0.001
Pre vs Full	−1.05	− 1.12, − 0.98	< 0.001
Interim vs Full	− 0.09	− 0.13, − 0.04	< 0.001

#### Secondary outcomes

The length of stay in the ED was shorter for all the GEDI intervention groups compared with pre-intervention (see Table 4). By comparison there was only the likelihood of a shorter hospital length of stay in the interim intervention period during GEDI hours, when compared to pre-GEDI.

**Table 4:** Secondary outcomes for GEDI Intervention

OUTCOME	Interim during GEDI hours Ratio (95% CI)	Interim outside GEDI hours Ratio (95% CI)	Full during GEDI hours Ratio (95% CI)	Full outside GEDI hours Ratio (95% CI)
^a^Shorter ED LoS	1.40	1.48	1.28	1.42
	(1.32, 1.48)	(1.42, 1.54)	(1.19, 1.38)	(1.33, 1.52)
^a^Shorter In-Hospital LoS	1.15	1.04	1.00	0.98
	(1.07, 1.23)	(0.99, 1.09)	(0.91, 1.11)	(0.90, 1.06)
^b^Risk of Death	0.32	0.52	1.01	0.77
	(0.08, 1.08)	(0.23, 1.14)	(0.23, 4.43)	(0.18, 3.22)
^a^Less same cause ED re-presentation within 28 days	1.13	1.04	1.21	1.19
	(0.89, 1.42)	(0.89, 1.22)	(0.88, 1.66)	(0.89, 1.58)
^a^Less any cause ED re-present within 28 days	1.18	1.06	1.21	1.10
	(1.02, 1.37)	(0.96, 1.18)	(0.99, 1.49)	(0.91, 1.32)

^a^ hazard ratio, ^b^ prevalence ratio, *NB* Reference for GEDI is pre-intervention

There was no clear difference in the risk of death for any of the GEDI periods when compared with pre-GEDI (see Table 4). We note the statistical power for this comparison is relatively low because deaths were rare. The likelihood of a shorter ED or hospital length of stay increased 2% for every 100 days of the trial.

Re-presentation for the same or any cause based on MDC was not altered by the intervention. The likelihood of re-presentation for other causes was increased if presenting in the interim GEDI period during working hours (see Table 4).

For the economic evaluation, we estimated a number of recursive model specifications to check for robustness and to test the sensitivity of the coefficient estimates. This analysis revealed that when compared to the pre-GEDI period, costs per hospital admission in both the full-GEDI and interim-GEDI period were lower (full-GEDI period = −$1469 [95% CI, $1105, $1834] and interim-GEDI period = −$1018 [95% CI, $709, $1326].

## Discussion

### Principal findings

In this pragmatic trial we aimed to control for a range of factors that previous studies have shown influence outcomes for older adults who present to the ED. The results indicate that the GEDI model increases the likelihood of discharge, decreases ED and, to some extent, hospital length of stay and costs, with no effect on same cause re-presentation or mortality. Older patients who presented in the months when the GEDI model was in place, and were subsequently admitted to hospital, spent 1 day less in hospital per admission, compared with patients presenting when no GEDI model was implemented and this resulted in cost saving. Other factors also influenced these outcomes with general changes in service provision over time increasing the likelihood of discharge directly from ED.

### Strengths and limitations of the study

This study capitalised on an opportunity to test the effect of a new model of service delivery over the course of its development and once the final version had been determined. Often new models of service delivery are not sustained [27]. Similarly, the final model tested in this intervention did not perform as well as the interim model (possibly representing the enthusiastic efforts of the innovative clinicians that began the change process) but it still outperformed the model employed prior to the commencement of the intervention.

While this pragmatic study used a pre-post design rather than a randomised controlled design to test the effect of the GEDI intervention, design features were incorporated to provide greater generalisability of the results. Our analysis included a variable to adjust for other changes that may have been occurring, in the study ED, over time (for example, maturation of the whole team or other interventions that were introduced to decrease ED length of stay). We also used the survival analysis taking into account major factors identified in the literature that affect patient outcomes for this cohort.

Functional decline and quality of life are important outcomes for this population. However, these variables are not routinely collected in our hospital and hence could not be examined here because of our retrospective study design.

### Comparison with other studies

There are a range of other models of care which aim to improve outcomes for older adults presenting to the ED. Some are outreach models or Hospital in the Nursing Home models in which ED or hospital clinicians travel to RACFs aiming to prevent transfer [15, 37–40]. These outreach models, unlike the GEDI model, may de-skill RACF staff and result in general medical practitioner disengagement. They may also be very expensive, although it is difficult to understand cost as few robust cost analyses have been undertaken [1]. Additionally, these models only work for the small proportion of older adults living in RACFs.

Other models focus on enhancing the care within the ED. The senior work up assessment and treatment (SWAT) model [41], the Triage and Rapid Elderly Assessment Team (TREAT) model [12], the Aged Care Services Emergency Team (ASET) Program [42], the GEDI WISE transitional care nurses [43] and a range of other models that combine some level of increased geriatric assessment and liaison with community services [44] have been described. Of these models only the first three report rigorous evaluative research outcomes. Unlike our GEDI model the SWAT [41] model, of increased senior medical officer involvement, did not improve overall ED length of stay but did improve length of stay on high volume days and for discharged patients. The TREAT [12] model which involved a highly skilled specialist team of consultant geriatricians and physicians, nurse practitioners and allied health staff, did reduce hospital admission but as there was no economic evaluation it is not possible to compare the costs of this service with the GEDI model. The GEDI WISE [43] transitional care model is the model most similar to GEDI and also resulted in decreased admissions. However, there is no specific physician champion in the GEDI WISE model and the increase in re-presentation seen in their model, but not seen in our GEDI evaluation, may be because of the important role played by the senior medical officer. The physician champion role, in this nurse-led model, is unique to GEDI and ensures there is multi-disciplinary involvement and that the care of older adults is seen as being as important as the care of other cohorts within the ED. In addition, the provision of post ED support to prevent re-presentations in the community dwelling cohort is dependent upon a supportive community-based health network. The ED physician champion plays a role in ensuring collaboration between the ED setting and community health services to provide continuity of client care.

### Meaning of the study

Focusing on improving the care of older adults in the ED and on strategies to prevent inappropriate ED presentation by older adults is of increasing importance in light of population ageing. Sinha and colleagues [17] identified the eight components of ED-based models of care for non-institutionalised older adults. The GEDI model was developed to include all of these components. A key finding of Sinha et al. [17] was that, “collaborative working practices are critical in model implementation and rely on the interpersonal skill sets of the clinicians delivering those initiatives and their ability to earn the trust and respect of their colleagues within and beyond the ED” (p. 680). Our findings suggest that it is possible to have advanced practice nurses, rather than nurse practitioners, in the GEDI model provided there is a strong physician-champion who supports, advises and works collaboratively with the senior nurse. This provides a relatively inexpensive yet effective model of care that reduces length of stay and increases discharge of older adults from the ED, when appropriate.

## Conclusions

There is a need for senior managers and policy makers to reconsider the models of care employed in ED service delivery for vulnerable groups. It is well accepted that emergency care for paediatric patients requires specialized resources including equipment, drugs, trained personnel, and facilities [45]. Yet for other vulnerable groups the provision of specialist care within the ED or increased training in a specialty for ED clinicians is not recognised. The results of this study suggest that having teams of emergency clinicians (doctors and nurses) with previous training and experience in geriatrics and community care, who are focused on streamlining the care of frail older adults in the ED, improves outcomes and cuts costs. Furthermore, these benefits can be sustained over time and while they may slightly decrease when the innovative team that effects change have moved to more senior roles, overall improvement remains. Finding solutions that maximise the outcomes for older people living in RACFs, who develop an acute illness, will involve greater inter-sectoral collaboration and require further research.

## Abbreviations

CEDRiC project: Care coordination through Emergency Department, Residential Aged Care and Primary Health Collaboration project
ED: Emergency Department
GEDI: Geriatric Emergency Department Intervention
HREC: Human Research Ethics Committee
PHN: Primary health network
RACF: Residential aged care facility
